# Phenotypes of Allo- and Autoimmune Antibody Responses to FVIII Characterized by Surface Plasmon Resonance

**DOI:** 10.1371/journal.pone.0061120

**Published:** 2013-05-08

**Authors:** Kenneth B. Lewis, Richard J. Hughes, Melinda S. Epstein, Neil C. Josephson, Christine L. Kempton, Craig M. Kessler, Nigel S. Key, Tom E. Howard, Rebecca Kruse-Jarres, Jeanne M. Lusher, Christopher E. Walsh, Raymond G. Watts, Ruth A. Ettinger, Kathleen P. Pratt, the PATH (Personalized Alternative Therapies for Haemophilia) Study Investigators

**Affiliations:** 1 Puget Sound Blood Center Research Institute, Seattle, Washington, United States of America; 2 Veterans Affairs Greater Los Angeles Healthcare System, Los Angeles, California, United States of America; 3 Division of Hematology, University of Washington, Seattle, Washington, United States of America; 4 Emory University, Atlanta, Georgia, United States of America; 5 Georgetown University, Washington, DC, United States of America; 6 University of North Carolina, Chapel Hill, North Carolina, United States of America; 7 Division of Hematology and Oncology, Department of Medicine, David Geffen School of Medicine at UCLA and Department of Pathology and Laboratory Medicine, Keck School of Medicine at USC, Los Angeles, California, United States of America; 8 Tulane University, New Orleans, Louisiana, United States of America; 9 Wayne State University, Detroit, Michigan, United States of America; 10 Mount Sinai School of Medicine, New York, New York, United States of America; 11 University of Alabama at Birmingham, Birmingham, Alabama, United States of America; National Cerebral and Cardiovascular Center, Japan

## Abstract

Evidence of antibody isotype/subtype switching may provide prognostic value regarding the state of immune responses to therapeutic proteins, e.g. anti-factor VIII (FVIII) antibodies that develop in many hemophilia A patients, clinically termed “inhibitors”. A sensitive, high- information-content surface plasmon resonance (SPR) assay has been developed to quantify IgG subtype distributions and the domain specificity of anti-drug antibodies. Plasma samples from 22 subjects with an allo- or auto-immune reaction to FVIII were analyzed. Pre-analytical treatment protocols were developed to minimize non-specific binding and specific matrix interference due to von Willebrand factor-FVIII interactions. The dynamic range for IgG quantification was 0.2–5 µg/ml (∼1–33 nM), allowing characterization of inhibitor-positive samples. Subtype-specific monoclonal antibodies were used to quantify the IgG subtype distribution of FVIII-specific antibodies. Most samples obtained from multiply-infused inhibitor subjects contained IgG4 antibodies. Several distinct phenotypes were assigned based on the IgG subtype distribution: IgG_1_, IgG_4_, IgG_1_ & IgG_4_, and IgG_1_, IgG_2_ & IgG_4_. An IgG_1_-only response was found in mild/moderate HA subjects during early FVIII infusions, and analysis of serial samples followed antibody class switching as several subjects’ immune responses developed. Competition studies utilizing a recombinant FVIII-C2 domain indicated 40–80% of FVIII-specific antibodies in most samples were directed against this domain.

## Introduction

The development of anti-FVIII allo-antibodies (“inhibitors”) occurs in a significant proportion of congenital Hemophilia A (HA) patients receiving exogenous FVIII, thereby rendering protein replacement therapy ineffective [1]. Additionally, anti-FVIII auto-antibody responses, though rare, can also occur, primarily in the elderly, postpartum or following traumatic injury. Allo antibodies develop as an anti-drug antibody response to FVIII infusions used to treat HA, and earlier detection and characterization of these responses may be useful to clinicians, *e.g.* as they tailor FVIII infusion schedules or consider immunosuppression regimes based on the perceived risk of a given patient developing a higher-titer response. In contrast, FVIII autoantibodies are virtually always diagnosed after they have reached a high titer, as testing is carried out after a non-hemophilic patient presents with unexplained bleeding and/or bruising. Clinical diagnosis of inhibitors is based on the Bethesda assay, a functional measurement of the inhibition of FVIII-mediated clotting of normal human plasma by antibodies in test plasma [2], [3]. An inhibitor titer of 1 Bethesda Unit (BU)/ml inhibits FVIII activity in normal pooled plasma by 50%. Non-inhibitory anti-FVIII antibodies are not detected by the Bethesda assay and quantification of inhibitors becomes unreliable when responses are <1 BU/ml; alternative assays are required to accurately quantify low-titer anti-FVIII antibodies. Although inhibitory antibodies are the primary concern when attempting to restore hemostatic function, both inhibitory and non-inhibitory antibodies provide information about the immunological state of a patient. A number of sensitive immunoassays have been developed to allow the screening of clinical samples for total (inhibitory+non-inhibitory) anti-FVIII antibodies and to provide complementary information to the Bethesda assay [4]–[9].

Early stages of alloimmune responses to FVIII include stimulation of helper T cells, which secrete cytokines leading to production of anti-FVIII antibodies by plasma cells, antibody class switching, affinity maturation, and generation of antibodies recognizing specific epitopes on the FVIII surface [10]. The complexity of these responses, for example the immunoglobulin isotypes and subtypes involved, the number of epitopes recognized, the clonality (polyclonal, oligoclonal, monoclonal) of the response, and the antibody affinities, provides important information as to the phenotypes of developing immune responses. Detailed characterization of the early stages of anti-drug antibody responses may provide information needed to design new clinical assays and may also indicate mechanisms leading to high-titer inhibitors versus immune tolerance (defined operationally for HA patients as having either no anti-FVIII antibodies or a low-titer response that does not seriously compromise hemostasis).

Comprehensive characterization of complex anti-FVIII antibody responses can be time- and resource intensive and numerous technical challenges, including inadequate sensitivity, exist. Surface Plasmon Resonance (SPR) offers a detection platform that is versatile, robust, and amenable to complex, multiplexed measurements of plasma samples. The relative speed with which SPR sensorgrams can be generated and analyzed also makes this technique suitable for medium- to high-throughput analysis of multiple samples. This report describes the use of an SPR assay to define phenotypes of allo- and autoimmune antibody responses based on antigen-specific IgG subclass distribution and epitope (FVIII domain) specificity. Plasma samples were collected from 18 HA and four acquired HA (autoimmune) patients with developing or persistent immune responses. Serial samples were collected from one young HA subject as he received initial FVIII infusions, and from one mild HA subject and two autoimmune HA subjects beginning with their initial inhibitor diagnosis. Although correlation of phenotypes with clinical outcomes is not definitive due to the small set of ADA-positive samples analyzed herein, the current study lays groundwork for analyzing plasma/serum samples from larger studies, including prospective studies. The stability and sensitivity of the SPR assay platform is demonstrated, and specific measurements containing clinically relevant information are identified, *e.g.* the quantitative distribution of antigen-specific IgG subtypes and the domain specificity of human anti-FVIII antibodies, specifically the fraction directed against the FVIII-C2 versus other domains.

## Materials and Methods

### Ethics Statement

This study was approved by the Seattle Children's Hospital IRB (SCH IRB#13018). Written informed consent was obtained from all adult subjects and from the next of kin, caretakers, or guardians on the behalf of the minors/children participants involved in this study, according to the principles expressed in the Declaration of Helsinki.

### Reagents

Expired Recombinate^tm^ (Baxter) was reconstituted as directed and used without further manipulation as the source of full-length human FVIII. Amino-terminally His_10_-tagged FVIII-C2 domain was produced as a soluble cytoplasmic protein in *E. coli* OrigamiB(DE3)pLysS (EMD Chemicals, Gibbstown, NJ). Caprylic acid, carboxy methyl dextran, 99.5% L-arginine and other reagents were from Sigma (St. Louis, MO). CM5 sensor chips, 1-ethyl-3-(3-dimethylaminopropyl) carbodiimide HCl (EDC), N-hydroxysuccinimide (NHS), ethanolamine, HBS-P+ buffer (10 mM HEPES, 150 mM NaCl, 0.05% (v/v) surfactant P20, pH 7.4) and sodium acetate pH 5.0 were from GE Healthcare Life Sciences (Piscataway, NJ).

### Antibodies

Mouse anti-human FVIII-A1 domain specific mAb (clone GMA-8004) was generously provided by Green Mountain Antibodies. An additional FVIII-C2 domain specific antibody (ESH4) was from American Diagnostica (Stamford, CT). Monoclonal anti-huIgG_1_ (clone HP6188) was obtained from Fitzgerald Industries International (Acton, MA). Anti-huIgG_2_ (clone HP6002), anti huIgG_3_ (clones HP6050 and HP6047), and anti-huIgG_4_ (clone HP6023) were from Southern Biotech (Birmingham, AL). Anti-huIgA (clone 8203) and anti-huIgM (clone 7408) were from Medix Biochemica (Finland). The human anti-FVIII-C2 mAb B02C11, both the IgG_4_ and Fab forms, were generously provided by Dr. M. Jacquemin [11]. Antibody concentrations were measured using a nominal extinction coefficient of ε^280 nm,0.1%^  = 1.38.

### Plasma Samples

Blood samples from subjects with HA and with autoimmune responses to FVIII (acquired HA) were collected as part of a cross-sectional study (NIH 1RC2HL101851) or were obtained from a Repository maintained by the Pratt laboratory. Plasma samples from subjects with and without a recently measured inhibitor titer in BU/ml were characterized using the SPR assay. Two types of samples were studied: sodium citrate anti-coagulated plasma (citrated plasma) and diluted heparin-anticoagulated plasma retained following isolation of peripheral blood mononuclear cells (“Ficoll plasma”).

### Pre-analytical Treatment

Pre-analytical treatment of plasma samples was performed using caprylic acid (CA) to precipitate non-IgG proteins and other interfering substances, including von Willebrand factor and hence baseline circulating FVIII (“CA treated plasma”). Citrated plasma samples (100–500 µL) were treated by mixing 1 part plasma with 2 parts 40 mM sodium acetate pH 4.0 and adding CA to a final concentration of 2.5% v/v (158 mM). Following 60 min incubation at room temperature with occasional mixing, samples were centrifuged for 5 minutes at 16,000×g to pellet the precipitate and filtered using a 0.2 µm Spin-X filter (Corning). The transparent filtrate was neutralized by adding 1 part to 9 parts 800 mM HEPES pH 8.0, 4 M NaCl and 5% carboxy methyl dextran. Ficoll plasma (typically 2–6 fold diluted) was treated similarly, however initial acidification was performed by adding 1 part to 9 parts 400 mM sodium acetate pH 4.0 to minimize further dilution. Nominal pre-analytical dilution factors were calculated for each sample.

### SPR Method

SPR measurements were carried out using a Biacore T-100 instrument (GE Healthcare Life Sciences) with binding measurements taken at 25°C. Murine anti-FVIII-A1 (GMA-8004) capture antibody was immobilized covalently onto a CM5 sensor chip from a 100 µg/ml solution in 10 mM sodium acetate pH 5.0 using a mixture of 0.4 M EDC and 0.1 M NHS. After immobilizing the capture antibody, the remaining active sites on the sensor chip were blocked by treatment with 1 M ethanolamine. A final immobilization signal of 9000 RU was targeted.

Binding experiments were performed in HBS-P^+^ containing 5 mM CaCl_2_ (HBS-P^+^/Ca^2+^). All injection and binding steps were performed at a slow flow rate (5 µl/min) to minimize FVIII, test plasma and secondary mAb consumption. FVIII (2000–3500 RU) was captured on the GMA-8004 antibody surface by injecting undiluted drug product for 300–600 sec. The dissociation of FVIII from this mAb was slow enough that the effect on RU signals measured at the report points was negligible (**Figure S1**). CA-treated plasma samples were injected for 300 sec followed by sequential 120 sec injections of 25–50 µg/ml secondary (isotype-specific) mAbs. Regeneration of the capture surface was achieved with three 20 sec injections of 2 M arginine pH 3.0 at 30 µl/min. To confirm that CA treatment did not alter the anti-FVIII IgG content of the test plasma, independent samples of untreated inhibitor negative HA plasma containing 1 µg/ml B02C11 (human IgG_4_) were prepared, CA treated, and the RU signals were compared.

To measure the fraction of the antibody response specific for the FVIII-C2 domain, paired plasma samples from four inhibitor subjects were tested by SPR with and without the addition of increasing concentrations of recombinant FVIII-C2 protein (the CA-treated plasma samples were added to either FVIII-C2 or the same volume of PBS as a negative control). Plasma samples were diluted first if necessary to bring the total anti-FVIII IgG titer below 5 µg/ml (∼33 nM). Samples were vortexed, centrifuged, and the supernatants stored at 4°C until analysis by SPR.

### Data Analysis

The SPR experiments were carried out under saturation binding conditions for the secondary mAbs to determine the maximum signal from each secondary mAb. This should correspond to stoichiometric binding of the secondary mAbs to the primary IgGs from plasma. Since the nominal molecular weights of human plasma anti-FVIII IgG and mouse anti-human IgG mAbs are comparable (∼150 kDa), the binding signal (RU) for both primary (binding of human anti-FVIII antibodies to the captured FVIII) and secondary (binding of subtype-specific mouse mAbs to human IgG captured from plasma) events should be directly comparable. Quantitative measurements (report points) of FVIII capture level, primary human IgG binding level, and secondary mAb binding levels were recorded 30 sec after the end of each sequential injection step.

Singly referenced binding curves were recorded as the signal from an active flow cell (with captured FVIII) minus the signal from a reference flow cell (without FVIII). Each assay sequence contained mAb B02C11 calibrators (0, 0.2, 1.0, 2.0, and 5.0 µg/ml prepared using CA-treated inhibitor negative HA plasma). Since the FVIII capture level declined slowly over the course of each sequence of samples (due to gradual degradation of the capture mAb following multiple regeneration cycles) and subsequent binding of plasma Abs and secondary mAbs scaled with the FVIII capture level, all binding signals were first normalized to a nominal capture level of 3000 RU FVIII. Calibrators and test samples were typically tested in blocks of 5 injections that were bracketed by a complete injection cycle in which assay buffer was substituted for the test sample. The average binding signals for the bracketing buffer injections were subtracted from the test sample signals to correct for minor signal variations due to incomplete regeneration and/or sensor degradation. Binding signals were converted from RU to µg/ml IgG using the secondary binding levels for the B02C11 calibrators. The ratios of the total cumulative secondary mAb binding signal to the primary human antibody binding signal were also calculated.

## Results

### Assay Performance

Acceptable assay performance was typically achieved for 100–150 cycles with a single sensor chip. FVIII capture capacity declined slowly, but this was not typically a limitation. A more significant limitation was a progressive increase in non-sample-specific secondary antibody binding signal, necessitating the frequent inclusion of bracketing injections of buffer before and after the injection of plasma samples. Therefore, subtraction of reference RU values sometimes caused apparent negative referenced binding signals for samples with very low measured RU binding signals, e.g. the % anti-IgG_2_ signals from several plasma samples (**Tables 1** **–** **3**). If the response (in RUs) of bracketing buffer injections was reproducible, sample signals were corrected by subtracting the mean signals from the bracketing buffer injections. If not, samples were retested using a new sensor chip.

**Table 1:** Antibody subtypes and estimated titers by SPR.

Subject	IgG1+2+3+4 (RU)^a^/polyclonal IgG (RU)	% IgG_1_	% IgG_2_	% IgG_3_	%IgG_4_	Total anti-FVIII IgG from SPR (µg/ml)
**Predominantly IgG_1_ Response**						
17A (n = 2) b	1.11(.02)^c^	95%(2%)	0%(1%)	−1%(0%)	5%(1%)	3.11(0.62)
17A+FVIII-C2	ND^d^	ND	ND	ND	ND	<0.2
N-008	0.98	104%	−6%	1%	1%	5.45
N-008+ FVIII-C2	1.01	106%	−10%	3%	0%	2.4
L-006-001	0.87	92%	4%	4%	0%	11.85
L-006-001+ FVIII-C2	0.83	88%	7%	5%	−1%	4.89
**Predominantly IgG_4_ Response**						
F-014 (n = 2)	1.26(.07)	16%(1%)	−1%(0%)	0%(1%)	85%(1%)	2.67(0.53)
F-014+ FVIII-C2(n = 2)	1.49(.13)	14%(1%)	−17%(0%)	2%(2%)	101%(3%)	1.09(0.18)
B-002	1.24	8%	7%	−1%	86%	2.42
B-002+ FVIII-C2	1.26	7%	7%	−1%	86%	2.38
A-002	1.16	4%	5%	−1%	92%	4.38
A-002+ FVIII-C2	1.24	1%	7%	−1%	93%	2.57
**Mixed IgG Subtype Response**						
G-004	1.13	43%	−4%	−1%	62%	9.1
G-004+ FVIII-C2	1.25	42%	−10%	−1%	69%	5.11
C-010	0.81	80%	−3%	−2%	25%	1.59
C-010+ FVIII-C2	0.82	55%	−3%	−2%	50%	0.9
D-006 (n = 3)	2.04(.4)	45%(3%)	−6%(7%)	1%(1%)	61%(3%)	1.53(0.41)
D-006+ FVIII-C2	ND	ND	ND	ND	ND	<0.2
L-025	1.12	72%	1%	−1%	28%	3.56
L-025+ FVIII-C2	1.15	64%	3%	−1%	34%	2.17
P-011	1.18	38%	1%	−1%	61%	18.29
P-011+ FVIII-C2	1.2	29%	−1%	−1%	73%	11.28
P-001	0.98	23%	3%	0%	75%	22.58
P-001+ FVIII-C2	1.02	34%	4%	−1%	63%	3.97
F-006	1.01	31%	8%	−2%	62%	24.94
F-006+ FVIII-C2	1.19	42%	6%	−2%	54%	6.46
A-008	1.09	41%	11%	−1%	49%	3.78
A-008+ FVIII-C2	1.12	36%	11%	−1%	54%	2.02
F-025 (n = 2)	2.19(.51)	30%(1%)	22%(4%)	−1%(0%)	50%(3%)	0.90(0.16)
F-025+ FVIII-C2	ND	ND	ND	ND	ND	<0.2
C-019	4.07	61%	−9%	−1%	49%	1.62
C-019+ FVIII-C2	1.25	63%	−8%	−3%	48%	1.52
C-028	0.94	19%	14%	−1%	68%	8.17
C-028+ FVIII-C2	0.74	16%	18%	−2%	69%	4.18
**Primary binding to FVIII signal (in RU) does not match summed IgG1+IgG2+IgG3+IgG4 signal (in RU)**						
H-001	0.22	56%	29%	−4%	19%	2.54
H-001+ FVIII-C2	0.15	56%	34%	−5%	15%	0.96
**Autoimmune Subjects**						
Q-011-001	0.96	79%	4%	−1%	18%	34.25
Q-011-001+ FVIII-C2	0.98	79%	4%	−1%	18%	33.19
Q-012-001 (n = 4)	1.02(.04)	6%(1%)	2%(4%)	−1%(0%)	94%(5%)	6.40(3.66)
Q-012-001+ FVIII-C2	1.23	2%	−4%	−1%	103%	2.78
Q-033 (n = 2)	0.75(.06)	82%(1%)	6%(1%)	−1%(0%)	13%(0%)	23.97
Q-033+ FVIII-C2	0.85	89%	2%	−1%	10%	11.39
Q-016 (n = 2)	0.96(.01)	23%(1%)	8%(1%)	−1%(0%)	70%(3%)	26.88
Q-016+ FVIII-C2 (n = 2)	1.02(.03)	23%(1%)	5%(2%)	−1%(0%)	72%(3%)	11.19(1.54)

a The ratios indicate the agreement between the summed SPR signals from the binding of secondary detection antibodies specific for IgG_1_, IgG_2_, IgG_3_ and IgG_4_ (numerator) to the initial SPR signal generated by the anti-FVIII antibodies in plasma that bound to the immobilized FVIII (denominator).

b Multiple measurements (n) were made when sufficient plasma was available.

c Standard deviations are reported for these experiments in parentheses.

d ND = Not Determined because the low total IgG titer made estimates of ratios and %Ig subtypes unreliable.

**Table 2:** Antibody subtypes and estimated titers by SPR.

Subject	FVIII-C2 competition assay	Time since inhibitor diagnosis	IgG1+2+3+4 (RU)^a/^polyclonal IgG (RU)	% IgG_1_	% IgG_2_	% IgG_3_	%IgG_4_	Total anti-FVIII IgG (µg/ml)
Q-011		day1	0.96	79%	4%	−1%	18%	34.25
		day4	0.96	71%	9%	−1%	22%	27.43
		4 wk	1.2	78%	5%	−1%	18%	22.49
		6 wk (n = 3)^b^	1.09(.30)^c^	69%(4%)	10%(1%)	−2%(1%)	23%(2%)	6.02(3.79)
		8 wk (n = 3)	1.17(.74)	72%(7%)	10%(2%)	−1%(3%)	19%(3%)	1.36(0.95)
		22 wk (n = 2)	0.97(.05)	89%(7%)	4%(2%)	−2%(2%)	9%(4%)	0.94(0.11)
		32 wk (n = 2)	1.04(.03)	83%(1%)	2%(0%)	−1%(0%)	15%(1%)	1.58(0.35)
Q-012		day1 (n = 4)	1.02(.04)	6%(1%)	2%(4%)	−1%(0%)	94%(5%)	6.40(3.66)
		day7	1	6%	5%	−1%	90%	>23.50
		day9 (n = 3)	0.99(.06)	6%(0%)	5%(1%)	1%(1%)	90%(0%)	8.90(4.14)
		day13 (n = 2)	0.99(.11)	7%(0%)	4%(0%)	0%(1%)	89%(1%)	6.72(1.97)
		26 wk	ND^d^	ND	ND	ND	ND	<0.2
		34 wk	ND	ND	ND	ND	ND	<0.2
		42 wk	ND	ND	ND	ND	ND	<0.2
17A	(−) FVIII-C2	1 wk	1	101%	0%	−1%	−1%	31.39
	(+) FVIII-C2		0.7	102%	0%	−1%	−1%	9.11
	(−) FVIII-C2	3 wk	1.05	101%	1%	−1%	0%	23.54
	(+) FVIII-C2		0.74	104%	−1%	−3%	−1%	1.42
	(−) FVIII-C2	51 wk	1.04(.01)	99%(0%)	1%(0%)	(1%)(0%)	1%(0%)	5.49(0.09)
	(+) FVIII-C2		ND	ND	ND	ND	ND	<0.2
	(−) FVIII-C2	5 yrs	1.11(.02)	95%(2%)	0%(1%)	−1%(0%)	5%(1%)	3.11(0.62)
	(+) FVIII-C2		ND	ND	ND	ND	ND	<0.2
L-006		1 wk	0.87	92%	4%	4%	0%	11.85
		5 wk (n = 2)	0.98(.01)	91%(2%)	2%(4%)	4%(0%)	3%(2%)	13.13(1.46)
		9 wk	0.98	85%	6%	2%	7%	11.97
		21 wk	ND	ND	ND	ND	ND	<1.09
		35 wk	ND	ND	ND	ND	ND	<0.99

a The ratios indicate the agreement between the summed SPR signals from the binding of secondary detection antibodies specific for IgG_1_, IgG_2_, IgG_3_ and IgG_4_ (numerator) to the initial SPR signal generated by the anti-FVIII antibodies in plasma that bound to the immobilized FVIII (denominator).

b Multiple measurements (n) were made when sufficient plasma was available.

c Standard deviations are reported for these experiments in parentheses.

d ND = Not Determined because the low total IgG titer made estimates of ratios and %Ig subtypes unreliable.

**Table 3:** Clinical data for subjects.

Subject	Age	HA Severity	Baseline FVIII	Peak Titer(BU/ml)^a^	Inhibitor Treatment History	Hemophilia Genotype(if known)
**Predominantly IgG1 Response**						
17A	24	mild	6–14%	250	ITI failed^b^	A2201P
N-008	2	moderate	3%	11	no ITI	14–21 del^c^
L-006	2	moderate	1%	87	ITI initiated	R2304C
**Predominantly IgG4 Response**						
F-014	19	severe	<1%	32	ITI partly successful	int-22 inv^d^
B-002	20	severe	<1%	667	ITI failed	9–11 del^e^
A-002	14	severe	<1%	256	ITI failed	not inversion^f^
**Mixed IgG Subtype Response**						
G-004	16	severe	<1%	1000+	no ITI	int-22 inv
C-010	27	severe	<1%	80	ITI partly successful	not inversion
D-006	10	severe	<1%	496	ITI failed	not inversion
L-025	35	severe	<1%	191	no ITI	not inversion
P-011	8	severe	<1%	1084.4	ITI failed	int-22 inv
P-001	12	severe	<1%	308.7	ITI failed	int-22 inv
F-006	27	severe	<0.25%	44	no ITI	int-22 inv
A-008	31	severe	<1%	86	ITI successful	int-22 inv
F-025	21	severe	<1%	43.8	ITI failed	int-22 inv
C-019	60	severe	<1%	336	ITI failed	int-22 inv
C-028	2	severe	<1%	96	ITI failed	not inversion
**Secondary and primary SPR binding signals (in RU) do not match**						
H-001	50	severe	<1%	742	no ITI	int-22 inv
**Autoimmune subjects**						
Q-011	77	autoimmune	normal	6	prednisone	autoimmune
Q-012	77	autoimmune	normal	2	prednisone	autoimmune
Q-033	79	autoimmune	normal	39	prednisone	autoimmune
Q-016	62	autoimmune	normal	20	prednisone	autoimmune

a BU/ml = Bethesda Units/milliliter;

b ITI = Immune Tolerance Induction;

c 14–21del = exons 14–21 deleted;

d int-22 inv = intron 22 inversion;

e 9–11del = exons 9–11 deleted;

f not inversion = not an intron-22 or intron-1 inversion mutation.

**Figure 1:**
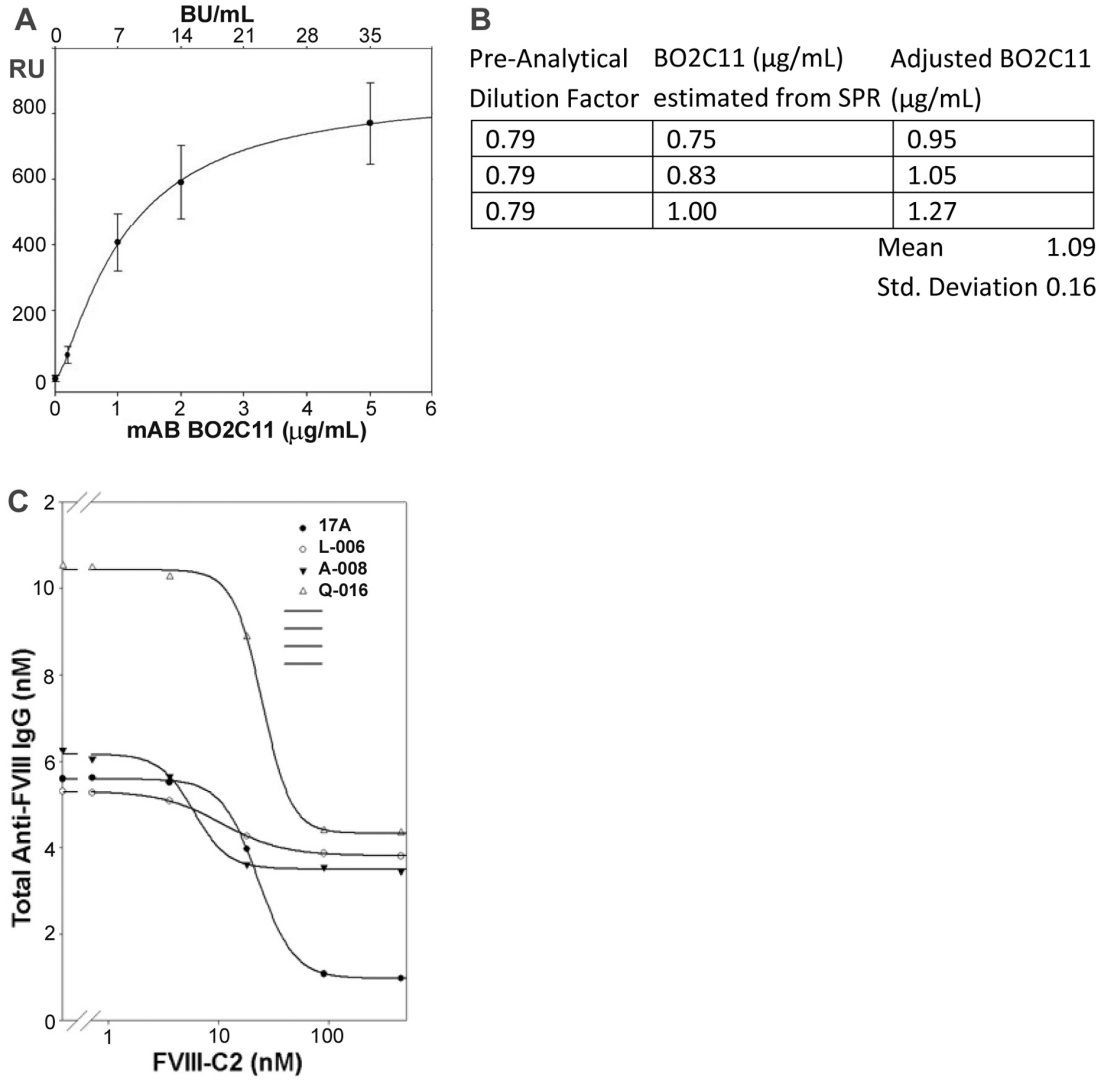
Characterization of plasma samples by SPR. A) Representative BO2C11 calibration curve obtained from 8 independent SPR runs in which this mAb was added to a FVIII- and inhibitor-negative plasma sample that was pretreated with CA. The plasma used in these experiments showed no evidence of anti-FVIII antibodies when tested by SPR using the FVIII-capture format (not shown). The final added BO2C11 concentrations are shown below the x-axis and the FVIII inhibitor titers indicated in Bethesda units (BU)/ml are based on the specific activity of BO2C11 = 7,000 BU/mg [11]. B) Spike recovery of independent samples in which 1 µg/ml B02C11 was added to a FVIII- and inhibitor-negative plasma sample that was subsequently diluted and treated with CA and then analyzed by SPR. The measured RU values were converted to concentrations in µg/ml (central column) based on calibration curves generated for BO2C11 as shown in Figure 1A. The adjusted BO2C11 concentration (third column) is the measured BO2C11 concentration corrected for the 0.79 preanalytical dilution factor C) Titration inhibition curves showing addition of increasing concentrations of recombinant FVIII-C2 to CA-treated plasma from 4 inhibitor-positive subjects. The sample from subject Q-016 was diluted first in order to bring the total anti-FVIII antibody titer below 5 µg/mL (33 nM). The FVIII-C2-specific antibody fraction was saturated above 100 nM FVIII-C2 in all 4 samples.

**Figure 2:**
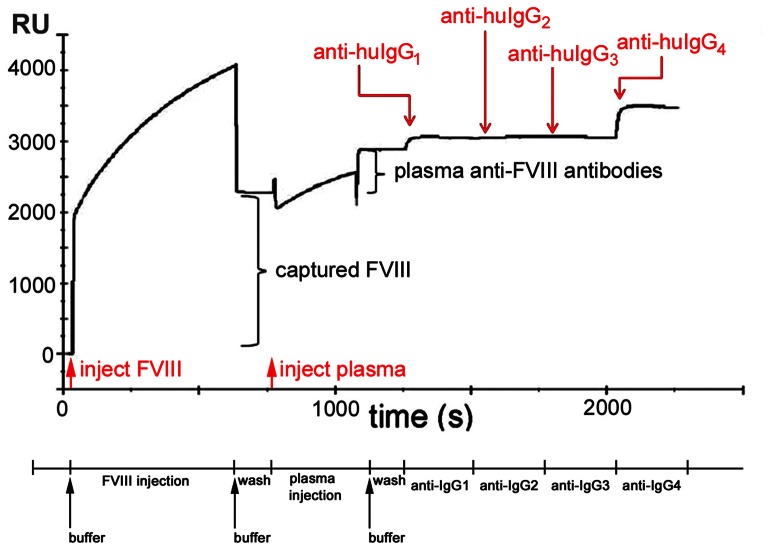
Representative binding curve (sensorgram) characterizing anti-FVIII antibodies in a human plasma sample. The sensorgram depicts the injection and capture of FVIII, the injection of test plasma and capture of human anti-FVIII antibodies, and sequential 120 sec injections and binding of mouse anti-human IgG_1_, anti-huIgG_2_, anti-huIgG_3_, and anti-huIgG_4_. The sequence of sample injections and wash steps is indicated below the x-axis, while the sensorgram shows the sequential contributions to the signal in RU due to (1) capture of injected FVIII (2277 RU); (2) attachment of antibodies from plasma to captured FVIII (839 RU); (3) attachment of anti-IgG_1_ secondary detection mAb to anti-FVIII antibodies that are subclass IgG_1_ (191 RU); (4) attachment of anti-IgG_2_ secondary mAb to anti-FVIII antibodies that are subclass IgG_2_ (75 RU); (5) (negligible) attachment of anti-IgG_3_ secondary mAb to anti-FVIII antibodies that are subclass IgG_3_ (−9 RU); (6) attachment of anti-IgG_4_ secondary mAb to anti-FVIII antibodies that are subclass IgG_4_ (558 RU). The baseline signal is set to 0 RU for the sensor surface with the immobilized capture antibody GMA-8004. Red arrows indicate injection points for samples and black arrows indicate injections points for buffer to initiate the intermediate wash steps. Note that a mismatch in the refractive index between the injected solution and the assay buffer results in a transient upward baseline shift in signal (during the injection of the FVIII in formulation buffer) or a transient downward shift (during the injection of the CA treated plasma sample). Since the secondary detection antibodies were diluted into assay buffer, such transient baseline shifts are less evident following the later injections. In order to accurately measure the binding signals following injections of FVIII, plasma antibodies, and detection antibodies, baseline measurements were taken 10 seconds prior to each injection, and the binding level measurements (report points) were taken 30 seconds after the end of each injection, when the sensor was again exposed to assay buffer and the refractive index shift was resolved. These report points were used to obtain the quantitative results summarized in Tables 1&2.

The use of affinity-captured FVIII antigen placed limits on the dynamic range of quantitative measurements. Although normalized calibration curves using the patient-derived inhibitory antibody BO2C11 were highly reproducible across multiple days and sensors (**Figure 1A**) the dynamic range for the SPR assay was narrow, with a range of quantification from 0.2 µg/ml (∼1 nM) to 5 µg/ml (∼33 nM). Below 0.2 µg/ml, signal to noise ratios were too low to obtain reliable information. BO2C11 binds to FVIII with an apparent dissociation constant K_D_ ∼ 2×10^−11^ mol L^−1^ and inhibits its pro-coagulant activity with a specific activity of ∼7,000 BU/mg [11]; these spike-recovery assays indicated the lower limit for detection of this unusually high-affinity neutralizing antibody by SPR was 0.2 µg/ml ∼1.4 BU/ml. Above 5 µg/ml, accurate concentration measurements could not be obtained due to saturation of the affinity-captured FVIII, but the IgG subtype distribution could still be measured. The spike-recovery experiment in which 1.0 µg/ml (∼7 nM) B02C11 was added to plasma from a HA subject that contained no FVIII or anti-FVIII antibody, and then measured before and after CA treatment, demonstrated a recovery of 109±16% (**Figure 1B**). As expected for B02C11 (human IgG_4_), this response was IgG_4_–restricted and the ratio of secondary (anti huIgG_1_+ anti huIgG_2_+ anti huIgG_3_+ anti-huIgG_4_ signals) to primary (human anti-FVIII antibodies from the test plasma) binding RU signals was close to stoichiometric (94±10%). In addition to satisfactory recovery of B02C11 following CA treatment, the behavior of independently treated and tested samples from a given subject, including both citrated plasma and Ficoll-treated heparin-anticoagulated plasma, was reproducible when assayed using different sensors and with different sample preparations. Once treated with CA, the samples remained stable for several weeks at 4°C. SPR of four plasma samples incubated with different concentrations of FVIII-C2 showed that in all cases, the competitive response (recombinant FVIII-C2 displacing FVIII-C2-specific antibodies) was saturated by >100 nM FVIII-C2 (**Figure 1C**). **Figure 2** depicts a representative binding curve for a plasma sample having a complex antibody phenotype with injection steps and report points annotated.

### HA Phenotypes

**Figure 3:**
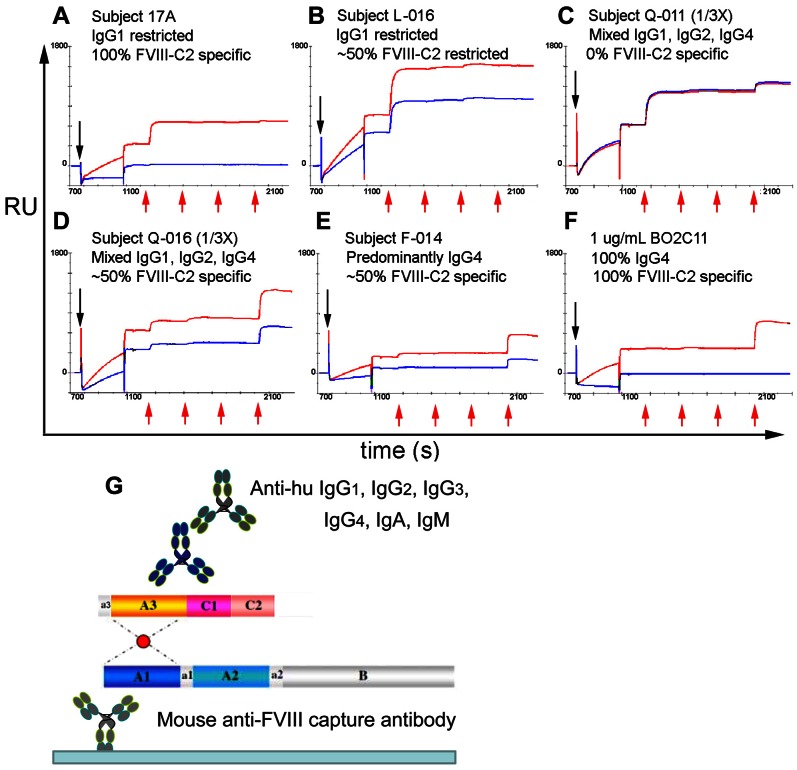
Binding curves from matched plasma samples with and without the addition of saturating (1 µM) recombinant FVIII-C2. (A–F) Black arrows pointing downwards indicate injection of CA treated plasma and red arrows indicate injections of anti-huIgG_1_, anti-huIgG_2_, anti-huIgG_3_ and anti-huIgG_4_. Injection of FVIII and its capture on mAb GMA-8004 (0–700 s) are not shown. (G) The Biosensor assay format is shown schematically.

Representative binding curves illustrating the range of phenotypic responses are shown in **Figure 3**. Each panel shows binding curves obtained in the presence and absence of excess (1 µM) FVIII-C2. Quantitative measurements (percent of the response derived from each human IgG subtype, total anti-FVIII IgG concentration (µg/ml), and the ratio of secondary to primary binding signal in %) obtained from the binding curves are tabulated in **Tables 1** **&** **2**. The total anti-FVIII IgG concentrations were corrected for pre analytical dilution factors and thus reflect the concentrations in undiluted plasma. Likewise values <0.2 µg/ml (the lower limit of quantification) are reported based on the assay dynamic range corrected for the sample dilution factor. The % IgG subtype values were not considered reliable when the total anti-FVIII IgG of a diluted or undiluted sample was <0.2 µg/ml so they are not reported in the tables. Almost every permutation (IgG subtype distribution, proportion of FVIII-C2 specific antibodies, and anti-FVIII IgG concentration) of phenotypic response was observed. Two subjects (B-002 and Q-011) demonstrated a complete lack of competition with FVIII-C2, whereas anti-FVIII antibodies in one sample from subject 17A were completely specific for FVIII-C2. However, the most common response was a mixed IgG subtype distribution with 40–80% FVIII-C2 specificity. For both the cross-sectional, single time point samples and the serial samples, no significant divergence between the total IgG subtype distribution and the FVIII-C2-specific IgG subtype distribution was observed. Three HA subjects (17A, N-008 and L-006) exhibited a predominantly IgG_1_ restricted response. Another three HA subjects (F-014, B-002 and A-002) exhibited predominantly IgG_4_-restricted responses, however detectable levels of other IgG subtypes were also observed. Samples from the four autoimmune HA subjects (Q-011, Q-012, Q-016 and Q-033) all exhibited complex mixtures of IgG_1_, IgG_2_ and IgG_4_ in addition to high total anti-FVIII IgG concentrations. In addition to testing with IgG subtype-specific secondary antibodies, the samples were screened with anti-IgA and anti-IgM secondary antibodies (not shown). No samples in this study exhibited an IgA or IgM response.

Serial samples were obtained from two of the autoimmune HA subjects (Q-011 and Q-012) and from two congenital HA subjects (L-006 and 17A) following initial presentation with an inhibitor. For samples from subjects Q-011 and Q-012 (**Table 2**), a progressive decrease in total anti-FVIII IgG concentrations was observed, with levels becoming undetectable six months after inhibitor diagnosis for Q-012, at which time the FVIII activity of the plasma was 36%. For the serial samples from moderate HA subject L-006, trace levels of IgG_3_ were observed in the earliest sample, and trace IgG_4_ was found in samples obtained one and two months later. Rituximab therapy subsequently resulted in a predictable decrease in his anti-FVIII IgG concentrations. For mild HA subject 17A, the response was IgG_1_-restricted throughout the first year following initial inhibitor detection, but a low-titer sample obtained 5 years later, during which time he received several additional FVIII infusions following a traumatic injury, showed partial IgG_4_ character. SPR measurements of a sample obtained from this subject 1–3 weeks after initial inhibitor detection indicated that ∼30% of the anti-FVIII antibodies bound to the FVIII C2 domain (estimated from RUs measured in the presence of saturating FVIII-C2 protein, **Table 2**) and samples obtained later in the course of this immune response exhibited nearly complete specificity for the C2 domain.

## Discussion

Inhibitor formation is a serious complication in the management of HA patients and more than thirty years of research has provided insight into humoral anti-FVIII immune responses, which often include IgG_4_ immunoglobulins [7], [12], [13]. The IgG_4_ subtype is frequently associated with chronic exposure to protein antigens [14]. Previous studies have demonstrated that anti-FVIII antibodies target multiple domains in FVIII [15]–[20]. Both the IgG subtype distribution and the complexity of the epitope distribution have been reported to be immunologically important, but they are not routinely measured [13], [17], [21], [22]. The present study describes a new methodology that allows quantification of anti-FVIII IgG isotype/subtype distributions and their gross domain specificity. (Future studies will examine specificity of IgGs for other domains besides FVIII-C2). The assay format is suitable for measurement of small (50–100 µL) volumes (*e.g.* residual samples from clinical assays) and for medium-throughput analysis of multiple samples. The ability to quantify the proportion of anti-FVIII IgGs with particular subtypes or domain specificities allows precise measurement of dynamic changes in both developing and resolving inhibitor responses.

### Assay Performance

The dynamic range of the SPR assay was from 0.2–5 µg/ml anti-FVIII antibody, corresponding to ∼1.4–35 BU/ml for the high-affinity neutralizing monoclonal antibody BO2C11 (**Figure 1A**). Subject Q-012 had an initial inhibitor titer of 2 BU/ml and the corresponding anti-FVIII antibody titer by SPR was ∼6.4 µg/ml (**Table 2**), indicating that a polyclonal inhibitor titer of ∼0.3 BU/ml could be detected. Thus the usable range of the SPR-based assay is similar to that of the Bethesda assay in detecting inhibitor responses, but the former can also be used to detect and characterize non-neutralizing antibody responses to FVIII.

The use of plasma and serum samples can introduce significant matrix interference into immunoassays due to competitive or nonspecific binding of proteins or other components. The SPR format is particularly sensitive to non-specific binding since the mass of bound protein (or other components in plasma or buffer) is measured, whether or not the interaction is specific. The SPR format, in which samples are tested sequentially rather than in parallel, also requires that an active sensor surface be regenerated between tests. Several factors contribute to the reproducibility and accuracy of the measurements reported herein: the use of CA-treatment as a pre-analytical step, the use of affinity-captured FVIII, and the use of monoclonal secondary antibodies to detect specific IgG subtypes.

Since inhibitory anti-FVIII antibodies may compete with vWF [23] for binding to FVIII [24], vWF was removed from test samples. Caprylic acid proved to be an effective pre-clearance step yielding samples with no residual vWF (**Figure S2**) and very low non-specific binding [25]. Like any pre analytical treatment, CA treatment may potentially alter the distribution of antibody populations in test samples [26]. The quantitative recovery of B02C11 from CA-treated samples and the highly reproducible behavior of test plasmas treated independently at different times suggest that CA treatment did not alter the antibody profiles. More exhaustive spike-recovery experiments were not possible due to the lack of additional purified FVIII-specific human antibodies. FVIII is a labile protein, so covalent immobilization followed by repeated assays in which it is exposed to plasma would not be feasible. The use of affinity-captured FVIII as the antigen, although it limits the dynamic range of quantitative measurements, allows a fresh antigen surface to be used for each testing cycle. The capture format allows FVIII to be immobilized directly from a solution while it is still in formulation buffer, alleviating potential problems with antigen instability due to dilution and buffer exchange. Additionally, it allows the assay to be performed with different sources of FVIII without having to optimize immobilization conditions individually. The Biacore T100 and similar instruments have multiple flow cells that can be simultaneously exposed to test samples, so this assay format could easily be adapted to carry out multiple parallel measurements, *e.g.* to simultaneously test antibody responses to different FVIII products.

Although a variety of domain-specific FVIII mouse mAbs are available, capture of FVIII via different domains was not compared rigorously, primarily due to the lack of a well-characterized polyclonal pooled control plasma sample. FVIII-A1 specificity has not been reported for neutralizing anti-FVIII antibodies, therefore an anti-A1 domain monoclonal antibody (GMA-8004) was chosen to capture FVIII from plasma. Low pH, high concentration arginine is an effective eluant for the affinity purification of antibodies [27], and it proved to be an effective regeneration solution for this capture antibody.

Since the nominal molecular weights of human and mouse IgG are similar, the use of monoclonal secondary antibodies with a defined 1∶1 binding stoichiometry provides additional information that is not obtainable with polyclonal secondary antibodies. For most test samples, the total cumulative binding signal from the secondary mAbs corresponded closely (80–120%) to the primary binding signal (**Tables 1** **&** **2**). This provided confidence that the measured IgG subtype distribution accurately reflected the FVIII-specific antibody response in the test plasma. The sample from subject H-001 was a notable exception (**Figure S3**). This sample exhibited a strong, partial FVIII-C2 specific response with a complex, mixed IgG_1_+IgG_2_+IgG_4_ profile. However, the cumulative secondary mAb signal accounted for only approximately 20% of the primary signal in RUs. The cause of this discrepancy (*i.e.* a non-IgG plasma component bound to captured FVIII) is unknown. All of the subtype-specific detection mAbs used in this study have been reported to be acceptable for detection of human IgG subtypes by ELISA [13], [28]–[30]. For H-001, an additional IgG_3_-specific secondary antibody (clone HP6047) also failed to detect a measurable IgG_3_ component to this anti-FVIII immune response. Although no samples from this study exhibited a strong IgG_3_ response, serial samples from L-006 reproducibly showed trace IgG_3_ content.

Both the quantity of monoclonal antibody GMA-8004 that can be covalently linked to the sensor and the relatively low concentration of the FVIII drug product used as the antigen source limit the ability to capture large quantities of FVIII and achieve mass transport limited conditions that are necessary to obtain accurate concentration measurements. Consequently there was some variability in the calibration curves, especially at the higher B02C11 concentrations. This necessitated the inclusion of a calibration curve for every sequence of samples tested (**Figure 1A**). Satisfactory and reproducible assay responses were obtained when test samples were diluted to 0.2–5 µg/ml (1.3–33 nM) total anti-FVIII antibody.

This was a preliminary study and suitable control samples with well defined polyclonal distributions of human anti-FVIII antibodies were not available to formally assess recovery after CA treatment, accuracy, and precision. However, a number of samples were tested multiple times over the course of assay development and routine sample testing. The results from these tests provide a measure of the reproducibility of this assay format. When multiple tests were performed, the number of tests (n = #) and the standard deviation of each measurement (#) are indicated in parentheses in Tables 1 and 2.

### HA Phenotypes

The Concerted Action on Neutralizing Antibodies in severe hemophilia A (CANAL) study reported that inhibitor development generally occurs after a median of 14 exposure days [31]. The present study enrolled HA subjects age 2 and above. Therefore, it was not surprising that class switching had already occurred in almost all of the inhibitor subjects. **Table 3** summarizes demographic and HA-related clinical information regarding the inhibitor-positive subjects. Consistent with previous studies [12], [13], [21], their immune responses to FVIII were dominated by IgG_1_ and IgG_4_, with minor IgG_2_ and/or IgG_3_ components observed in higher-titer, more complex responses. The most notable responses from this panel of samples were for subjects 17A (mild HA, infused multiple times), N-008 (moderately severe HA, inhibitor detected after his 9^th^ FVIII infusion, sample obtained 2 months later), and L-006 (severe HA, serial samples obtained following initial inhibitor detection after 11 FVIII infusions), who all demonstrated IgG_1_-restricted responses. Several other mild/moderate HA subjects had no anti-FVIII antibodies detectable by SPR. The IgG_1_-restricted responses may simply reflect limited exposure to FVIII, as mild/moderate HA patients generally receive FVIII infusions only to treat severe bleeding or during surgery.

At the other end of the spectrum, the autoimmune HA subjects exhibited a complex response with respect to the IgG subtype distribution, total anti-FVIII IgG concentration and apparent ratio of anti-FVIII IgG to inhibitory antibodies (BU/ml). The complexity of these responses suggests they were flare-ups of previous sub-clinical autoimmune responses to FVIII. Prescott *et al* (1997), measuring only inhibitory antibodies, observed that the autoimmune responses were less complex than alloimmune HA responses [17]. However, they noted that although inhibitors primarily targeted only a few FVIII domains, their specificity for additional FVIII domains was indicated by immunoprecipitation experiments, suggesting that non-inhibitory antibodies were also present. The present study also evaluated the fractions of antibodies specific for the FVIII-C2 domain versus those specific for other FVIII domains in the autoimmune subjects. Samples from a larger number of subjects, and utilizing other FVIII fragments or hybrid/mutant FVIII proteins for competition assays, will be required to further characterize and quantify the epitope specificity of anti-FVIII antibodies by SPR.

In general, solution phase competition experiments with matched samples yield unequivocal, easily interpretable results. This was observed in the present study, measuring the FVIII-C2 specific IgG response, and in earlier studies that identified the domain specificity of inhibitory antibodies using the Bethesda assay [17]. Alternative approaches, based on competition binding to immobilized FVIII between mAbs with known specificity and anti-FVIII antibodies in plasma [32]–[34], use of hybrid porcine/human FVIII proteins [35], [36], FVIII mutagenesis [36], phage display [37] FVIII peptide-binding assays [38]–[40] and Luminex technology [41] have also been described.

As noted above, competition studies with FVIII-C2 protein consistently demonstrated that specificity for this domain was not linked to specific IgG subtypes. This result is consistent with the observations of Kessel *et al.* [37] and also with the concept that IgG class switching occurs after epitope specificity has been determined. Some measurement of the clonality of FVIII-specific antibodies would be a valuable metric to gauge the complexity of inhibitor responses. However, both IgG class switching and the prevalence of IgG_4_ in the samples complicate the definition of clonality. The desired information may actually be the clonality of FVIII-specific precursor B-cells prior to class switching. It is important to note that in IgG_4_-dominated responses, ELISAs using anti-kappa and anti-lambda chain secondary antibodies to address clonality of the responses may be misleading since circulating IgG_4_ molecules are functionally “bi-clonal” due to exchange of half-IgG_4_ molecules with other (non FVIII-specific) IgG_4_ antibodies [42]–[44].

### Conclusions

The SPR method described herein is an easily adaptable assay format with which to characterize anti-FVIII antibody responses. The assay sensitivity is satisfactory to characterize most inhibitors detectable using the Bethesda assay, as well as samples containing anti-FVIII antibodies (neutralizing+non-neutralizing) with concentrations >0.2 µg/ml. Several observations were notable: As has already been reported, the IgG_4_ subtype was commonly observed, typically in mixed subtype responses. However, three HA subjects with inhibitor responses (2 emerging, 1 chronic) demonstrated IgG_1_-restricted responses. Also, most subjects exhibited partial FVIII-C2 specificity. Autoimmune subjects exhibited complex responses involving multiple IgG subtypes, multiple domain specificities, high total anti-FVIII antibody concentrations, and an apparently high ratio of total to inhibitory anti FVIII IgG. The present study analyzed plasma samples from 22 inhibitor subjects, including serial samples from two HA subjects with a recently diagnosed inhibitor and two acquired HA subjects following initial detection of their inhibitor. Future studies analyzing a larger set of plasma samples will compare the anti-FVIII total antibody and antibody-subtype titers estimated from SPR sensorgrams with titers derived from quantitative ELISA assays [13]. Such larger studies will also establish the relative sensitivity of SPR, ELISA and Bethesda assays in detecting and characterizing anti-FVIII antibody responses. The SPR platform described herein is a promising approach to carry out future prospective studies of FVIII inhibitors and other anti-drug antibody responses. Because of the small plasma volumes required and the quick assay turnaround time, this method is especially suitable for batch analysis of multiple samples, e.g. central laboratory characterization of antibody responses to FVIII or other clinically important antigens.

## Supporting Information

Figure S1 **Binding kinetics of FVIII captured on the anti-FVIII-A1 domain antibody GMA-8004.** **A.** MAb GMA-8004 was immobilized on a CM5 chip as described in Methods. Recombinate was then injected and the binding kinetics were measured at flow rates 5 µl/min and 30 µl/min. X-offset and y-offset were performed using the Biacore software to match the end of the association phase for the 5 µl/min and 30 µl/min curves. **B**. Magnified view of the dissociation over 30 min, which was ∼10 RU at 5 µl/min (compared to the initial binding signal of 3215 RU) vs. ∼5 RU at 30 µl/min (compared to the initial binding signal of 865 RU). At both flow rates the total dissociation over 30 min was <1% of the initial signal in RUs. Note that the capture times were not adjusted to yield matching capture levels at the different flow rates so the amount of captured FVIII is lower at the lower flow rate.
(TIF)

Figure S2 **ELISA assays showing VWF in serially diluted Untreated and CA-treated plasma and serum samples.** No VWF was detected in the CA-treated samples.
(TIF)

Figure S3 **Binding curves for subject H-001 obtained in the presence and absence of excess (1** **µM) FVIII-C2.** Quantitative measurements (percent of the response derived from each human IgG subtype, total anti-FVIII IgG concentration (µg/ml), and the ratio of secondary to primary binding signal in %) obtained from the binding curves are tabulated in Table 1.
(TIF)

Table S1 **Subjects and samples.**
(DOC)

Supplementary File S1 A detailed description of FVIII dissociation kinetics from capture antibody GMA-8004 is provided and the preanalytical treatment of plasma to remove vWF is described.
(DOC)
