# Supplementary figures and images for: Phenotypes of Allo- and Autoimmune Antibody Responses to FVIII Characterized by Surface Plasmon Resonance
Source: PLoS One. 2013 May 8;8(5):e61120. doi: 10.1371/journal.pone.0061120 (PMC3648518; doi:10.1371/journal.pone.0061120)

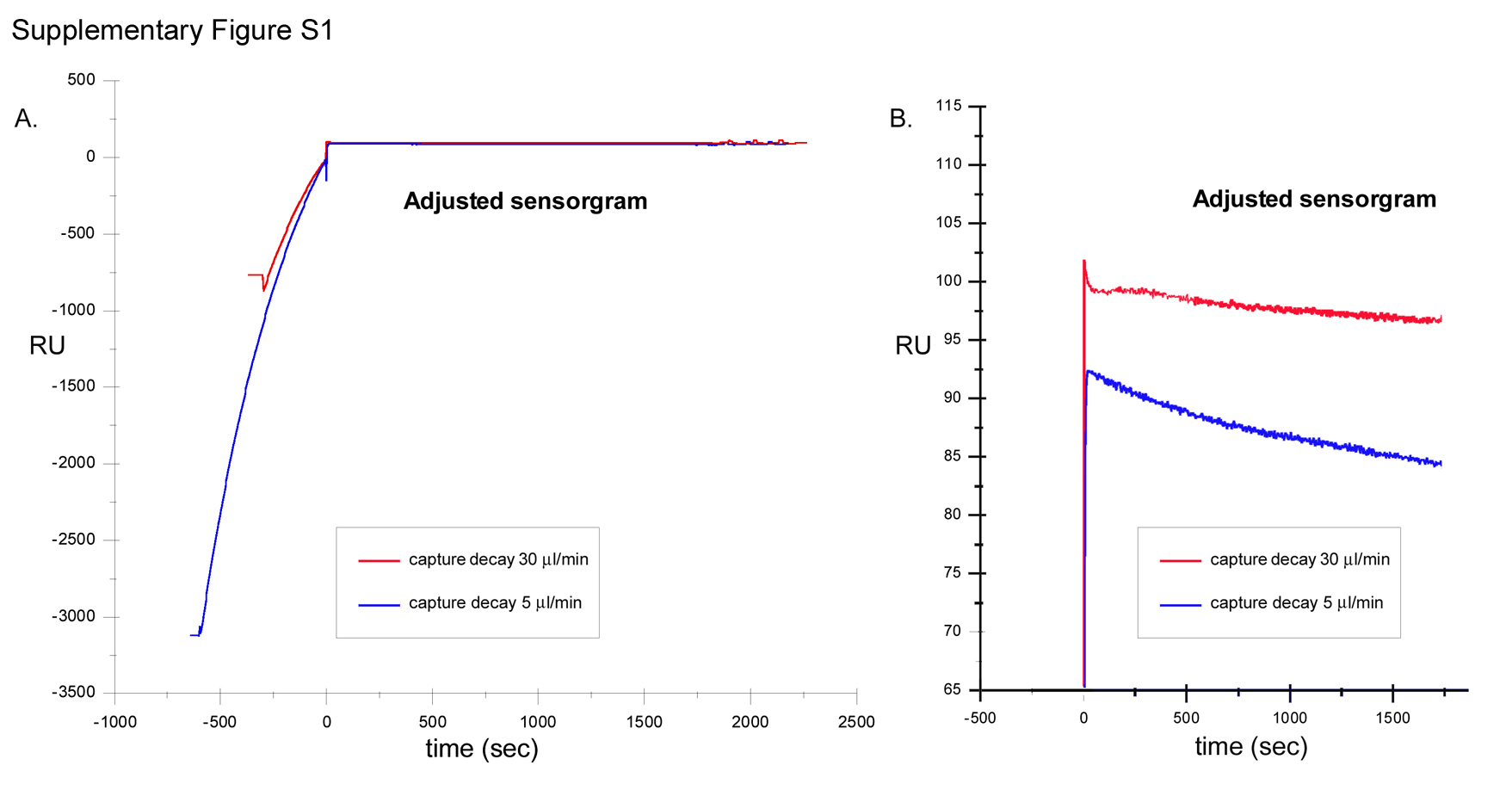

Supplement: Figure S1 — Binding kinetics of FVIII captured on the anti-FVIII-A1 domain antibody GMA-8004. A. MAb GMA-8004 was immobilized on a CM5 chip as described in Methods. Recombinate was then injected and the binding kinetics were measured at flow rates 5 µl/min and 30 µl/min. X-offset and y-offset were performed using the Biacore software to match the end of the association phase for the 5 µl/min and 30 µl/min curves. B. Magnified view of the dissociation over 30 min, which was ∼10 RU at 5 µl/min (compared to the initial binding signal of 3215 RU) vs. ∼5 RU at 30 µl/min (compared to the initial binding signal of 865 RU). At both flow rates the total dissociation over 30 min was <1% of the initial signal in RUs. Note that the capture times were not adjusted to yield matching capture levels at the different flow rates so the amount of captured FVIII is lower at the lower flow rate. (TIF) [file pone.0061120.s001.tif]

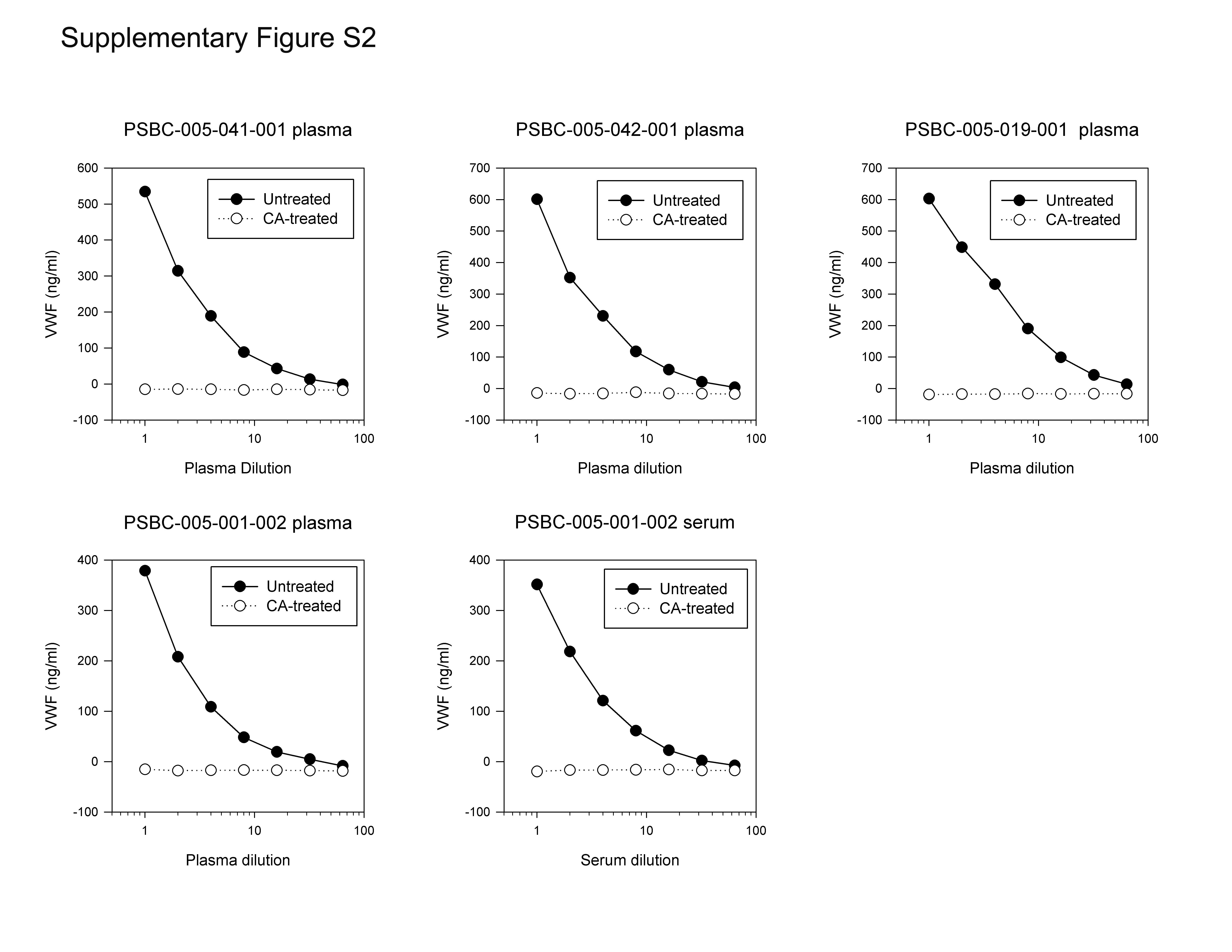

Supplement: Figure S2 — ELISA assays showing VWF in serially diluted Untreated and CA-treated plasma and serum samples. No VWF was detected in the CA-treated samples. (TIF) [file pone.0061120.s002.tif]

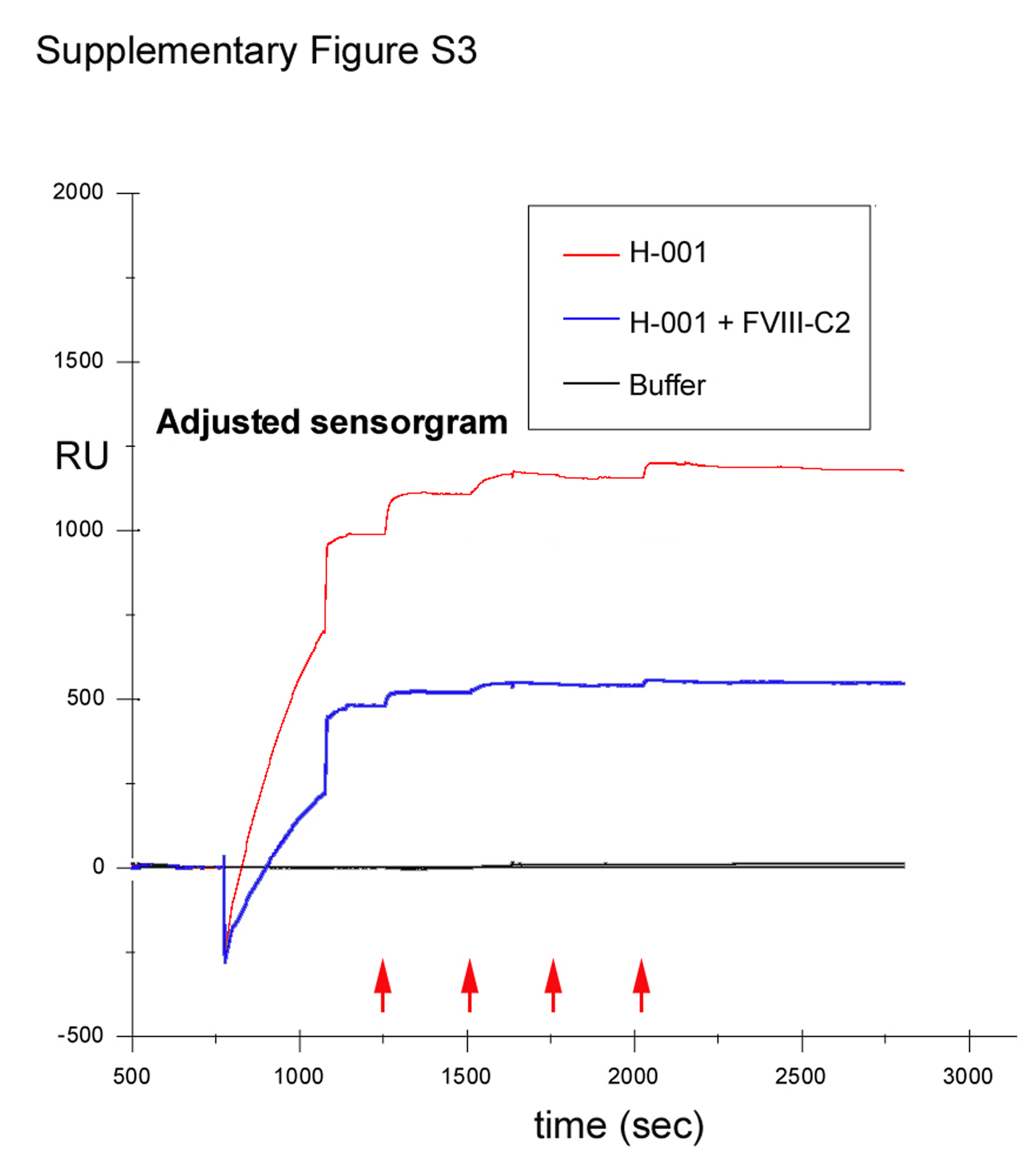

Supplement: Figure S3 — Binding curves for subject H-001 obtained in the presence and absence of excess (1 µM) FVIII-C2. Quantitative measurements (percent of the response derived from each human IgG subtype, total anti-FVIII IgG concentration (µg/ml), and the ratio of secondary to primary binding signal in %) obtained from the binding curves are tabulated in Table 1. (TIF) [file pone.0061120.s003.tif]
