# Supplementary material for: Phenotypes of Allo- and Autoimmune Antibody Responses to FVIII Characterized by Surface Plasmon Resonance
Source: PLoS One. 2013 May 8;8(5):e61120. doi: 10.1371/journal.pone.0061120 (PMC3648518; doi:10.1371/journal.pone.0061120)
Supplement: Table S1 — Subjects and samples. (DOC) [file pone.0061120.s004.doc]

| **Sample ID** | **Sample Type** | **Normal or HA** | **Fresh or frozen** |
| --- | --- | --- | --- |
| PSBC-005-041-001 | Citrate Plasma | Normal | Frozen |
| PSBC-005-042-001 | Citrate Plasma | Normal | Frozen |
| PSBC-005-019-001 | Citrate Plasma | Severe HA | Frozen |
| PSBC-005-001-002 | Citrate Plasma | Normal | Fresh |
| PSBC-005-001-002 | Serum | Normal | Fresh |
