# Supplementary material for: Phenotypes of Allo- and Autoimmune Antibody Responses to FVIII Characterized by Surface Plasmon Resonance
Source: PLoS One. 2013 May 8;8(5):e61120. doi: 10.1371/journal.pone.0061120 (PMC3648518; doi:10.1371/journal.pone.0061120)
Supplement: Supplementary File S1 — A detailed description of FVIII dissociation kinetics from capture antibody GMA-8004 is provided and the preanalytical treatment of plasma to remove vWF is described. (DOC) [file pone.0061120.s005.doc]

**SUPPLEMENTARY DATA**

*Capture and dissociation of FVIII using anti-A1 domain mAb GMA-8004*

The dissociation rate of Recombinate captured on GMA-8004 was measured at two flow rates, 5 µl/min and 30 µl/min, to determine if the dissociation was sufficiently slow to choose this as a capture antibody for FVIII. Flow cell #1 (un-conjugated) on the Biacore T100 was used as the reference flow cell. HBS EP Biacore BR-1006-69 Lot 2027942/67, a CM5 sensor chip (lot 10040595) and 70% glycerol for normalization (Biacore lot 123403) were from GE Healthcare Life Sciences. Recombinate was from Bayer. SPR runs were carried out by injecting Recombinate and measuring the binding kinetics over 30 min at flow rates 5ul/min and 30 ul/min. X-offset and y-offset were performed using the Biacore software to match the end of the association phase for the 5 µl/min and 30 µl/min curves (**Supplementary Figure S1**).

*Removal of vWF by CA treatment of plasma*

Pre-analytical treatment of plasma or serum samples was performed using caprylic acid (CA) as described under Methods. ELISA assays (see below) were carried out for a representative subset of samples (**Supplementary Table S1**) to ascertain whether CA treatment indeed removed all residual vWF. VWF was undetectable after CA treatment of normal citrated plasma, severe HA citrated plasma, normal serum, and in both fresh and frozen samples (**Supplementary Figure S2**).

*VWF ELISA method:* Polyclonal rabbit anti-human vWF Code A0082 (Dako, Carpinteria, CA) was diluted to 1 μg/ml in 0.01 M phosphate buffer, 0.15 M NaCl, pH 7.2. Diluted antibody (100 μl) was added to each well of NUNC Maxisorp 96 ELISA plates and incubated overnight at 4^o^C. Plates were then washed 5X in 300 μl 1X D-PBS containing 0.05% Tween 20 (wash buffer). Blocking buffer was prepared by diluting 5X assay diluent (eBiosciences, San Diego, CA) to 1X in MilliQ water. Blocking Buffer, 200 μl per well was added, and the plates were incubated for 3 hrs at room temperature. Serial dilutions (2X to 64X) into 20 mM HEPES, 150 mM NaCl, 0.05% Tween 80, pH 7.4 (Dilution Buffer) of untreated and CA treated plasma/serum samples were prepared. Untreated plasma samples were first diluted 30X to match the dilution of CA treatment prior to serial dilutions. Serial dilutions of purified vWF FVIII free (Haematologic Technologies Inc., Burlington, VT) (15.6-1000 ng/ml) were prepared to generate a standard curve. Plates were then washed 5X in wash buffer, 100 μl samples and standards were added to each plate and incubated for 2 hrs at room temperature. Plates were then washed 5X in wash buffer, peroxidase-conjugated rabbit anti-human vWF Code P0226 (Dako) was diluted 1:8000 in Dilution Buffer and 100 μl was added to each well and incubated for 1 hr at room temperature. Wells were washed 5X in Wash Buffer, 100 μl of Super Aquablue ELISA substrate (eBiosciences) was added to each well, and A_405_ was read on a Molecular Devices SpectraMax M5 ELISA plate reader. The amount of vWF in the plasma/serum samples was extrapolated from the standard curve using SoftMax Pro 5.2 software.
